# Why, what, who, when and how? Hand hygiene measured in India?

**DOI:** 10.1186/2047-2994-4-S1-P297

**Published:** 2015-06-16

**Authors:** J Hemalatha, D Sureshkumar

**Affiliations:** 1 Infectious Disease Education & Reserach Foundation, Chennai, India

## Introduction

Hand hygiene (HH) is the single most important factor in the prevention of health care-acquired infections. Comprehensive monitoring of hand hygiene compliance is considered integral parts of a successful hospital infection control program. However, measuring adherence to hand hygiene guidelines is resource intensive and complicated by lack of standardized methodology.

## Objectives

Our aim was to survey the methodologies used to measure hand hygiene guidelines across different Indian hospitals.

## Methods

The survey was conducted in person among infection control professionals working in different Indian hospitals (one per institution) using questionnaire developed based on WHO hand hygiene guidelines during their participation in international infection control conference held in India during the month of March 2015.

## Results

Infection control professionals representing 26 Indian hospitals (different types) participated in the survey. In India HH was measured mainly due to quality imitative (11/26 42.30%) or part of accreditation (10/26 -38.46%) requirement. The ICPs monitor HH majority of the time (24/26 92.30%), they focus mainly on monitoring adherence to 5 HH moments (15/26 -57.69%) and monitor during the day time (22/26 -84.61%). Once in a month (12/26-46.15%) and direct observation 22/26 (84.61%) was the preferred mode of monitoring in India.

## Conclusion

Majority of Indian hospitals monitor compliance to HH guidelines, however comprehensive monitoring of all aspects of HH was missing in many of the hospitals. Clearly, further research to develop efficient, reliable, and comprehensive methods for monitoring hand hygiene compliance is urgently required in India.

## Disclosure of interest

None declared.

